# *Plasmodium falciparum* clearance in clinical studies of artesunate-amodiaquine and comparator treatments in sub-Saharan Africa, 1999–2009

**DOI:** 10.1186/1475-2875-13-114

**Published:** 2014-03-25

**Authors:** Julien Zwang, Grant Dorsey, Andreas Mårtensson, Umberto d’Alessandro, Jean-Louis Ndiaye, Corine Karema, Abdoulaye Djimde, Philippe Brasseur, Sodiomon B Sirima, Piero Olliaro

**Affiliations:** 1 Drugs for Neglected Diseases Initiative (DNDi), Geneva, Switzerland; 2 Department of Medicine, University of California, San Francisco, CA, USA; 3 Division of Global Health (IHCAR), Department of Public Health Sciences, Karolinska Institutet, Stockholm, Sweden; 4 Disease Control and Elimination Theme, Medical Research Council Unit, Banjul, The Gambia; 5 Department of Parasitology, Faculty of Medicine, Cheikh Anta Diop University, Dakar, Sénégal; 6 Malaria & Other Parasitic Diseases Division-RBC, Ministry of Health, Rugenge, Kigali, Rwanda; 7 Malaria Research and Training Center, Department of Epidemiology of Parasitic Diseases, Faculty of Medicine and Pharmacy, University of Science, Techniques and Technology of Bamako, Bamako, Mali; 8 Institut de Recherche pour le Développement (IRD) Unité mixte de Recherche 198, Dakar BP 1386, Sénégal; 9 Centre National de Recherche et de Formation sur le Paludisme, Ministère de la Santé, Ouagadougou, Burkina Faso; 10 UNICEF/UNDP/WB/WHO Special Programme for Research & Training in Tropical Diseases (TDR), Geneva, Switzerland; 11 Centre for Tropical Medicine and Vaccinology, Nuffield Department of Medicine, University of Oxford, Churchill Hospital, Oxford OX3 7LJ, UK

## Abstract

**Background:**

Artemisinin-based combination therapy (ACT) is the recommended first-line therapy for uncomplicated *Plasmodium falciparum* malaria worldwide but decreased artemisinin susceptibility, phenotypically characterized as slow parasite clearance time (PCT), has now been reported in Southeast Asia. This makes it all too important to measure the dynamics of parasite clearance in African patients treated with ACT over time, to understand trends and detect changes early enough to intervene

**Methods:**

Individual patient data from 27 clinical trials of artesunate-amodiaquine (ASAQ) *vs* comparators conducted between 1999 and 2009 were analysed for parasite clearance on modified intent-to-treat (ITT) basis.

**Results:**

Overall 15,017 patients treated for uncomplicated *P. falciparum* malaria at 44 sites in 20 sub-Saharan African countries were included in the analysis; 51% (n=7,660) *vs* 49% (n=7,357) were treated with ASAQ and comparator treatments, respectively. Seventy-seven per cent (77%) were children under six years of age. The proportion of the patients treated with ASAQ with persistent parasitaemia on Day 2 was 8.6%, and 1.5% on Day 3. Risk factor for not clearing parasites on Day 2 and Day 3 calculated by multivariate logistic regression with random effect on site and controlling for treatment were: high parasitaemia before treatment was (adjusted risk ratios (AOR) 2.12, 95% CI 1.91-2.35, AOR 2.43, 95% CI 1.98-3.00, respectively); non-ACT treatment (p=0.001, for all comparisons). Anaemia (p=0.001) was an additional factor for Day 2 and young age (p=0.005) for Day 3.

In patients treated with ASAQ in studies who had complete parasitaemia data every 24 hours up to Day 3 and additionally Day 7, the parasite reduction ratio was 93.9% by Day 1 and 99.9% by Day 2. Using the median parasitaemia before treatment (p0=27,125 μL) and a fitted model, the predicted PCT (pPCT = 3.614*ln (p0) – 6.135, r² = 0.94) in ASAQ recipients was 31 hours.

**Conclusion:**

Within the period covered by these studies, rapid *Plasmodium falciparum* clearance continues to be achieved in Sub-Saharan African patients treated with ACT, and in particular with ASAQ. The prediction formula for parasite clearance time could be a pragmatic tool for studies with binary outcomes and once-daily sampling, both for research and monitoring purposes.

## Background

Delayed *Plasmodium falciparum* response to artemisinin is reported in Southeast Asia, and may spread to other, more intensely malaria-endemic areas. Artemisinin compounds contribute rapid parasite killing and faster clearance to artemisinin-containing combination therapy (ACT). Artemisinin tolerance/resistance manifests itself with slower parasite clearance, while ACT remain generally effective both clinically and parasitologically [1-4]. Genomic studies are underway, which have generated so far two sets of putative markers of artemisinin resistance - K13-propeller polymorphism [5], and a SNP in the gene encoding a DNA repair protein RAD5 on chromosome 13 (and possibly also on chromosome 10) [6]. An *in vitro* assay (Ring Stage Assay) has also focused on the very early phases of the ring-stage parasite [7]. However, as of today, in the clinic as well as in the field the parasite clearance time (PCT) and its related clinical phenotype (delayed PCT) remain the best practical surrogates of artemisinin *in vivo* resistance [8]; the problem is that the frequent sampling (every six or eight hours) required to measure PCT accurately and to estimate the parasite clearance half-life [9] is practically difficult even in research settings. Easier methods are needed for both research and routine purposes. For example, failure to clear by Day 3 (72 hours post-treatment start) is proposed as a simple and accurate predictor of treatment failure [10], requiring a single time-point and thus limiting the workload in resource-limited settings.

Applying these and other measures both retrospectively and prospectively to commonly-used ACT will help understand trends and detect changes early enough to prompt effective responses to contain the spread of resistance. Artesunate-amodiaquine (ASAQ) is the second most commonly used ACT in the world (the first-line treatment in 22 out of 44 sub-Saharan African countries); more than 200 million treatments of ASAQ Winthrop have been distributed in Africa since the medication became available in 2007 [11-13]. While ASAQ is generally effective, the decreasing efficacy of AQ single-agent treatment have been reported in some sub-Saharan African settings [14,15]. Should artemisinin resistance occur there, it is expected to result soon in treatment failures, particularly in subjects with partial immunity, such as children in high-transmission areas, or patients of all ages in low-transmission areas. Monitoring ASAQ efficacy, particularly in these groups, has therefore become all the more important.

Greater understanding of factors influencing parasite clearance is crucial, and requires the analysis of pooled data from individual patient records. Therefore, this study collected and analysed a large sample (over 15,000 patients) of individual patient data (of whom approximately three-quarters were children under six years of age) from clinical trials conducted in sub-Saharan Africa between 1999 and 2009.

Studies such as this will help provide the foundations for further analyses of parasite clearance trends across sub-Saharan malaria-endemic countries.

## Methods

### Study sites, design and patients

The database was constructed from studies identified through a systematic review of clinical trials and personal contacts. To be included, efficacy or tolerability monitoring studies had to have been conducted in sub-Saharan Africa including any formulation of ASAQ to any other single or combination treatment of uncomplicated falciparum malaria (non-severe or non hyperparasitaemic) with follow-up of at least 28 days. For the studies meeting these criteria, investigators were contacted to provide individual patient data and the datasets received were examined for inclusion [16].

### Study endpoints

Out of the 27 studies included (conducted between 1999 and 2009), three were multi-country studies [17-19], two studies compared ASAQ fixed and loose combination [20-22] and three studies were non-comparative (in Sierra Leone [23] and Senegal [24,25]). Details of the studies can be found elsewhere [26-40]. There was one unpublished study conducted in RDC (Cohuet, unpublished). The analysis of parasite clearance was by modified intent-to-treat (ITT), including all participants who were randomized and received the study medications. The primary endpoint in all the studies included in the analysis was parasitological efficacy, except one study about tolerability [22]. Follow-up ceased at the time of parasitological failure (either primary or recurrence), loss to follow-up, protocol violation and no data were recorded thereafter. Parasitaemia was recorded at enrolment (Day 0) and post-treatment from Day 1 through Day 28, but sampling schedules varied across studies during the first seven days, with Day 1 or Day 2 not recorded in all. Consecutive parasite slide results (Day 0, Day 1, Day 2, Day 3, Day 7) were available at 18 sites from five randomized controlled trials (RCT) [17-19,26,27].

### Definitions and analyses

Table 1 summarizes the measures and analyses conducted. The risks of delayed parasite clearance by Day 2 and Day 3 and parasite clearance failure were analysed as binary variables using age (continuous in years), baseline parasitaemia (log transformed) as continuous variables, anaemia (binary variable, haemoglobin < 10 g/dL), study year (continuous) and study (categorical). The parasite reduction ratio on Day (n) was defined as: parasitaemia before treatment/parasitaemia on Day (n).

**Table 1:** Definitions

**Measure**	**Definition**	**Population**	**Analysis**
Parasite clearance	Patients’ conversion from a positive to a negative parasite slide within 7 days post-treatment start (independent of whether it was sustained throughout the entire duration of follow-up or there was a recurrent episode).	All studies, whether comparative or not (n = 15,017)	Observed median time of parasite clearance
Delayed parasite clearance	- Parasitaemia positive on		Prevalence, risks by multivariate analysis
	o Day 2		
	o Day 3		
Parasite clearance failure	- Day 2 parasitaemia > pre-treatment parasitaemia		
	- Day 3 parasitaemia >25% pre-treatment parasitaemia		
	- Consecutive positive slides up to Day 7		
Parasite reduction ratio	Relative difference (referent: parasite before treatment)	ASAQ groups (n = 2,355) where parasite densities were measured once daily from Days 0 to 3 and Day 7	Reduction rate
Predicted time of parasite clearance	Modelling the time of parasite clearance		Predict the time of parasite clearance using the parasitaemia before treatment and the proportions of positive patients

The curve of the proportions of patients remaining parasitaemic during follow-up was fitted using logistic regression to give an estimate for clearance time. The fitted model for the logit of the proportion of parasitaemic patients over time and expressed in hours from the logistic regression:

$$\begin{array}{l} p\left(\text{day}\right)=\text{Logit}\;\left(\text{constant}–a*\left(\text{day}+1\right)\right)\\ \text{with}\;p\left(0\right)=\text{Logit}\;\left(\text{constant}\right)\\ \text{pPCT}\;\left(\text{hour}\right)=\left(\text{Logit}\;\left(-\text{constant}/a\right)–1\right)*24 \end{array}$$

In order to simplify the calculations of the predicted parasite clearance time ((pPCT), for a patient or a group of patients using the median time parasite clearance) the patient population was classed into ten pre-treatment parasite densities categories. A logistic model was fitted for each group and the corresponding predicted PCT was calculated, and once the results of predicted PCT for the ten groups were obtained, a simple logarithmic model was fitted with the aggregated results.

The risks, presented as adjusted risks ratios (AOR), were assessed by logistic multivariate analysis with random effect on the site in an attempt to account for potential statistical heterogeneity while controlling for age (continuous), parasitaemia before treatment (log-transformed), anaemia (binary), and year (continuous). Categorical data were compared using the Chi-square or the Fisher exact test as appropriate. The Spearman test was used to analyse the relationship between clearance reduction and pre-treatment parasitaemia. Confidence intervals (CI) were calculated at 95% and statistical significance was set at p-value <0.05.

### Ethical issues

All studies had been approved by the relevant ethics and institution review committees as reported in the individual papers.

### ASAQ treatment regimens

The majority of the patients were treated with individually formulated AS and AQ. The target dose was AS 12 mg/kg over three days and AQ 30 mg/kg over three days except in Uganda where AQ was given at 25 mg/kg (Day 0: 10 mg/kg, Day 1: 10 mg/kg, Day 2: 5 mg/kg). The loose combinations of ASAQ were dosed based on body weight, while in four studies the fixed dose combination (FDC) ASAQ was based on age and weight range [19-22]. The FDC was also given either once or twice a day [19].

ASAQ FDC (Coarsucam™ Winthrop® Sanofi Aventis); AS 25 mg/AQ 67.5 mg one tab/day for three days in children 5–8.9 kg; AS 50 mg/AQ 135 mg one tab/day for three days in children 9–17.9 kg; AS 100 mg/AQ 270 mg one tab/day for three days in children 18–35.9 kg.

### Comparator treatment regimens

i. ACT: artemether-lumefantrine (AL)(20 mg artemether/120 mg lumefantrine) given according to weight as one (5–14 kg), two (15–24 kg), three (25–34 kg), and four (≥35 kg) tablets given twice daily co-administrated with fat for three days; Coartem™, Novartis); dihydroartemisinin-piperaquine (DP) was given once daily over three days, at the standard dosage of 2.25 mg/kg and 18 mg/kg of dihydroartemisinin-piperaquine, respectively, rounded up to the nearest half tablet (two formulations were used: 20 mg dihydroartemisinin + 160 mg piperaquine and 40 mg dihydroartemisinin +20 mg piperaquine, Eurartesim™, Sigma-Tau, and in Rwanda, Artekin™, Holley); AS + sulphadoxine- pyrimethamine (SP)(AS 4 mg/kg/day; SP 25 mg/kg of sulphadoxine and 1.25 mg/kg of pyrimethamine administered in a co-formulated tablet as a single dose);

ii. Non-ACT: AQ + SP (AQ 10 mg/kg/day for three days and SP 25 mg/kg of sulphadoxine and 1.25 mg/kg of pyrimethamine administered in a co-formulated tablet (SP) as a single dose); chloroquine (CQ)(25 mg/kg over three days) and SP; AQ monotherapy (10 mg/kg/day for three days); AS monotherapy (AS 12 mg/kg over five days).

## Results

**Figure 1:**
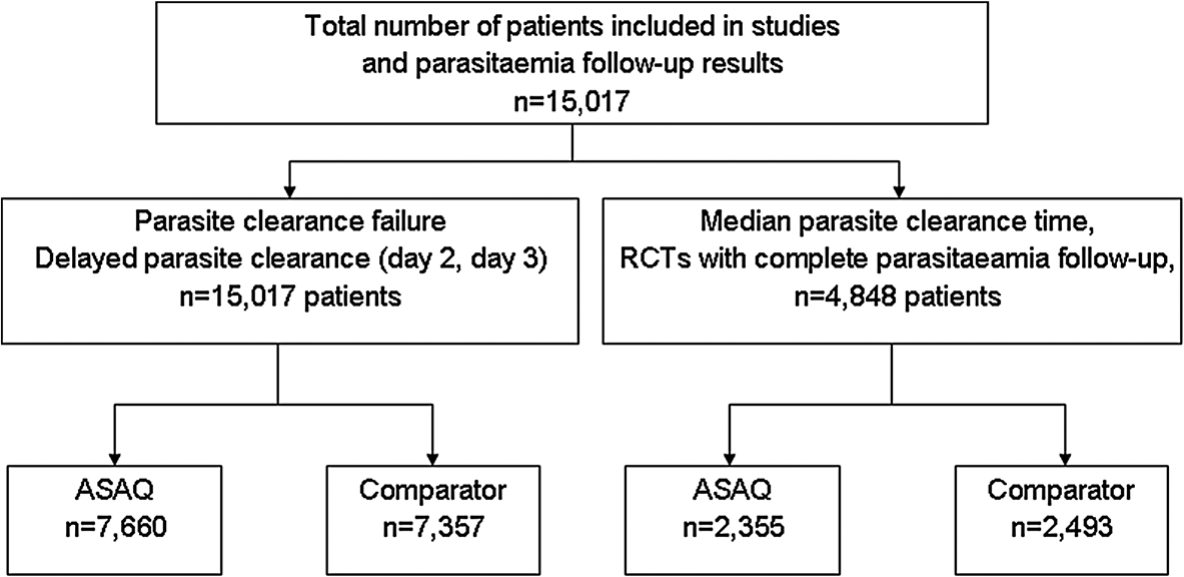
Flow chart, number of patients with parasitaemia results and daily results from Day 0 to Day 3 and Day 7.

Overall 15,017 patients treated for uncomplicated *P. falciparum* malaria at 44 sites in 20 sub-Saharan African countries were included in the analysis (Figure 1). The proportion of patients treated with ASAQ was 51% (n = 7,660) *vs* 49% (n = 7,357) on comparator treatments; 31% (4,848/15,017) of the patients had complete parasitaemia data every 24 hours up to Day 3 and then Day 7 (2,355 in ASAQ and 3,493 in comparator groups). Overall, the proportion of children under six years of age was 77%, and the overall geometric mean parasitaemia pre-treatment was 16,964/μL (Table 2).

**Table 2:** Patients at enrolment

**Year**	**Site**	**Total**	**Under six years old**		**Treatment**		**Parasitaemia**	
			**n**	**%**	**ASAQ (n)**	**Comparator (n)**	**μL**	**Anaemia (%)**
2004	Angola-Caala 2004	137	137	100	69	68	29,180	28
2003	Angola-Kuito 2003	187	187	100	97	90	19,216	61
2008	Burkina faso - Nanoro 2008	810	810	100	295	515	20,831	83
2005	Burkina Faso Puytenga 2005	890	824	93	890	NA	15,210	76
2006	Cameroon 2006	166	79	48	110	56	24,627	8
2004	Congo-Kindamba 2004	298	298	100	101	197	25,890	64
2008	Gabon - 2008	226	226	100	80	146	22,265	69
1999	Gabon 1999	220	119	54	110	110	22,537	NA
2004	Guinee-Dabola 2004	220	220	100	110	110	37,493	73
1999	Kenya 1999	400	346	87	200	200	31,095	61
2009	Kenya 2009	54	0	0	54	NA	12,181	2
2009	Liberia 2009	1300	302	23	648	652	1,340	11
2006	Madagascar 2006	179	76	42	119	60	9,159	19
2004	Mali Bancouna 2004	753	714	95	252	501	15,468	44
2006	Mali Bougoula 2006	203	134	66	135	68	22,183	51
2008	Mozambique - Manhica 2008	420	420	100	210	210	39,023	62
2008	Nigeria - Afokang 2008	261	261	100	92	169	18,361	72
2008	Nigeria - Pamol 2008	233	233	100	82	151	18,177	73
2003	RDC Boende 2003	279	279	100	136	143	24,170	57
2002	Rwanda Mashesha 2002	122	122	100	61	61	14,729	45
2004	Rwanda Mashesha 2004	269	269	100	89	180	20,454	39
2002	Rwanda Rukara 2002	95	95	100	49	46	25,855	40
2004	Rwanda Rukara 2004	270	269	100	89	181	36,868	31
2002	Rwanda-Kicukiro 2002	91	91	100	48	43	13,457	31
2004	Rwanda-Kicukiro 2004	223	223	100	74	149	36,515	28
2000-5	Sen-Djembeye 2000-5	137	18	13	137		25,992	8
1999	Senegal 1999	321	123	38	160	161	40,386	NA
2006	Senegal 2006	392	11	3	264	128	7,991	47
2000-5	Sen-Mlomp 2000-5	723	74	10	723	NA	46,328	3
2000-5	Sen-Oussouye 2000-5	208	106	51	208	NA	19,142	NA
2004	Sierra Leone Kailahun 2004	126	126	100	126	NA	27,116	56
2003	South Sudan Nuba 2003	161	161	100	80	81	21,964	31
2003	Sudan Malakal 2003	269	269	100	134	135	22,881	86
2008	Uganda - Mbarara 2008	319	319	100	160	159	23,431	51
2003	Uganda Amudat 2003	212	212	100	106	106	18,410	49
2004	Uganda Tororo 2004	541	508	94	194	347	18502	71%
2005	Uganda Tororo 2005	408	388	95	204	204	21,480	40
2004	Uganda-Apac-2004	542	516	95	174	368	11,701	64
2004	Uganda-Arua-2004	534	509	95	174	360	23,927	63
2003	Uganda-Jinja-2003	543	370	68	189	354	34,337	40
2006	Uganda-Kampala-2006	730	358	49	242	488	10,581	11
2008	Zambia - Ndola 2008	245	245	100	85	160	36,401	85
2002	Zanzibar Kivunge 2002	297	296	100	148	149	16,500	78
2002	Zanzibar Micheweni 2002	105	105	100	54	51	16,777	87
	Total	15017	11550	77	7660	7357	16,964	50

Legend: NA, non-applicable.

### Parasitaemia on Day 2 and Day 3

Delayed parasite clearance, defined as the proportion of patients still parasitaemic on Day 2 or Day 3 (analysed as a binary variable) under ASAQ treatment in comparative and non-comparative trials was analysed in 44 sub-Saharan African sites over the period 1999–2009.

The proportion of patients on ASAQ who were still parasitaemic on Day 2 was 8.6% (603/7,020, 95% CI 7.9-9.3%) and ranged from 1.0% in Burkina Faso-Nanoro (2008) and Congo-Kindamba (2004) to 57.1% in DRC-Boende in 2003 (Additional file 1: Table S1). Using multivariate logistic regression with random effect on site and controlling for treatment, the risk factors for a patient to remain positive on Day 2 were higher parasitaemia before treatment (AOR 2.12, 95% CI 1.91-2.35, p = 0.001) and anaemia (AOR 1.22, 95% CI 1.07-1.38, p = 0.001); no significant difference in the risk of being parasitaemic on Day 2 was detected in RCT comparing ASAQ to other ACT: AL (p = 0.245), DP (p = 0.762), AS + SP (p = 0.291), whereas patients treated with non-ACT were at higher risk: AQ + SP (AOR 14.53, 95% CI 11.36-18.59, p = 0.001), CQ + SP (AOR 20.10, 95% CI 15.07-26.82, p = 0.001), or AQ (AOR 21.63, 95% CI 12.73-36.75, p = 0.001).

**Figure 2:**
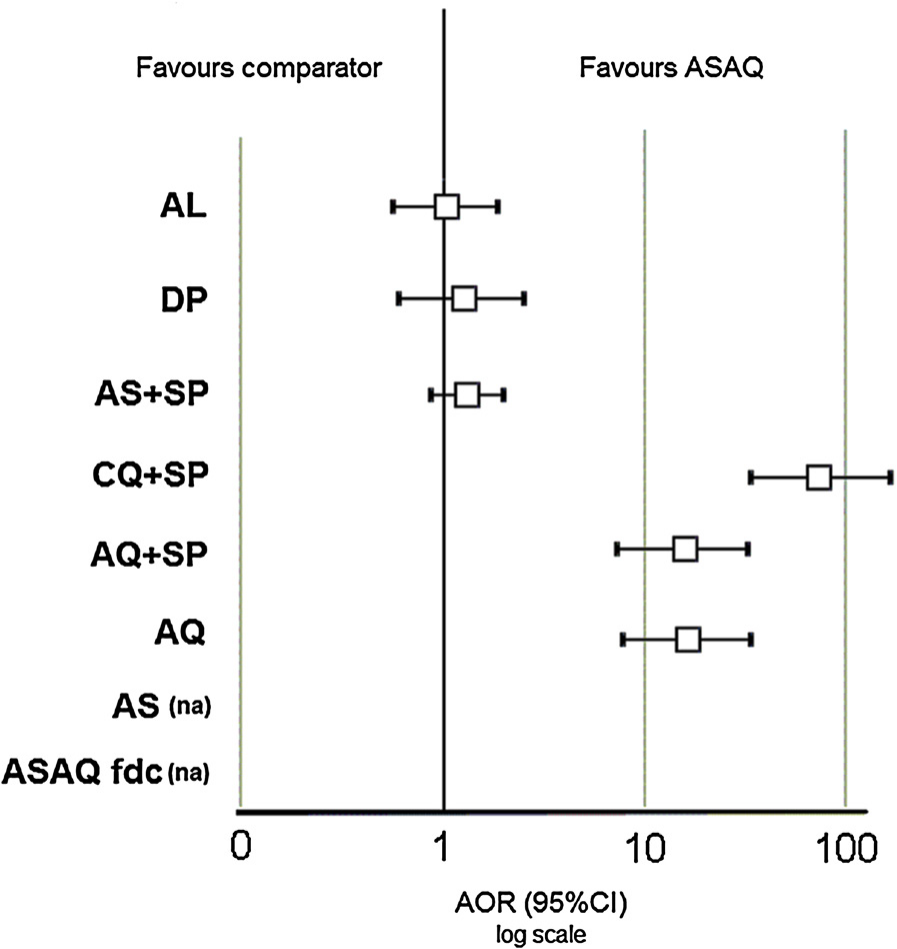
**Delayed parasitaemia (Day 3) forest plot, artesunate/amodiaquine *vs *comparator treatments.** AL: artemether-lumefantrine; AOR: adjusted odds ratio using multivariate logistic regression with random effects; AQ: amodiaquine; AS: artesunate; CQ: chloroquine; DP: dihydroartemisinin-piperaquine; fdc: fixed dose combination; na: not applicable; SP: sulphadoxine-pyrimethamine.

The proportion of patients treated with ASAQ who were still parasitaemic on Day 3 was 1.5% (116/7,550, 95% CI 1.2-1.8%, of whom 44 in RDC-Boende), ranging from 0% in many various sites across sub-Saharan Africa to 55.9% in DRC-Boende. Using multivariate logistic analysis with random effects on sites, younger patients (AOR 0.94, 95% CI 0.90-0.98, p = 0.005) and patients with higher parasitaemia at enrolment (AOR 2.43, 95% CI 1.98-3.00, p = 0.001) were at higher risk of remaining parasitaemic on Day 3. Compared to ASAQ, the risk of being parasitaemic on Day 3 was higher for patients treated with a non-ACT: AQ + SP (AOR 15.70, 95% CI 7.43-33.16, p = 0.001), AQ (AOR 16.38, 95% CI 7.82-34.34, p = 0.001), CQ + SP (AOR 72.56, 95% CI 33.70-156.23, p = 0.001), while no significant difference was detected with other ACT (AL, p = 0.993; DP, p = 0.525; AS + SP, p = 0.190) (Figure 2).

### Parasite clearance failure

Overall, patients treated with an ACT were at lower risk of parasite clearance failure by Day 7 (AOR 0.03, 95% CI 0.01-0.12, p = 0.001, stratified by site) compared to non-ACT (Table 3). The proportion of patients treated with ASAQ who had a parasite clearance failure was 0.2% (15/7,550, 95% CI 0.1-0.3%), mostly from one site (RDC-Boende). Using multivariate analysis with random effect on site, higher parasitaemia at enrolment (AOR 2.31, 95% CI 1.60-3.35, p = 0.001) was the only risk detected for parasite clearance failure.

**Table 3:** Proportion of patients with a parasite clearance failure by Day 7 and by treatment groups

**Treatment**	**Parasite clearance failure (number)**	**Total number of patients**	**Parasite clearance failure**		
			**Proportion %**	**95% CI lower bound**	**95% CI upper bound**
ASAQ	15	7,660	0.2	0.1	0.3
AL	3	2,391	0.1	0.0	0.4
DP	1	1,132	0.1	0.0	0.5
AS + SP	26	1,005	2.6	1.7	3.8
AQ	23	621	3.7	3.7	7.4
AQ + SP	9	1,257	0.7	0.3	1.3
AS	0	252	0.0	0.0	1.5
CQ + SP	78	699	11.2	8.9	13.7
Total	153	15,017	1.0	0.9	1.2

### Parasite reduction ratio

Overall, in studies treating with ASAQ and recording daily parasitaemia (n = 2,355), the parasite reduction ratio (parasitaemia before treatment/parasitaemia on day (n)) was 93.9% by Day 1 and 99.9% by Day 2 (Table 4). The parasite reduction ratio ranged on Day 1 from 77.1% in Mozambique to 99.2% in Kenya with a significant correlation between clearance reduction on Day 1 and pre-treatment parasitaemia (r = 0.098, p = 0.001, Spearman test, individual data).

**Table 4:** Clearance reduction, artesunate/amodiaquine groups

**Country site**	**Parasitaemia, geometric mean**	**N**	**Parasite reduction ratio (individual data)**			
			**d1/d0**	**d2/d0**	**d3/d0**	**d7/d0**
Burkina faso - Nanoro 2008	21,185	295	97.8%	100.0%	100.0%	100.0%
Cameroon 2006	24,129	110	97.0%	100.0%	100.0%	100.0%
Gabon - Fougamou 2008	21,117	80	98.6%	100.0%	100.0%	100.0%
Gabon 1999	20,867	108	98.2%	100.0%	100.0%	100.0%
Kenya 1999	30,040	199	94.5%	99.9%	100.0%	100.0%
Kenya 2009	12,193	54	99.2%	100.0%	100.0%	100.0%
Madagascar 2006	9,648	119	98.9%	100.0%	100.0%	100.0%
Mali Bougoula 2006	23,744	135	93.8%	99.9%	100.0%	100.0%
Mozambique - Manhica 2008	35,924	210	77.1%	99.8%	100.0%	100.0%
Nigeria - Afokang 2008	20,326	92	96.3%	99.7%	100.0%	100.0%
Nigeria - Pamol 2008	17,329	82	97.9%	99.8%	100.0%	100.0%
Senegal 1999	41,753	160	89.5%	99.8%	100.0%	100.0%
Senegal 2006	20,201	264	91.4%	99.7%	100.0%	100.0%
Uganda - Mbarara 2008	27,262	160	98.4%	100.0%	100.0%	100.0%
Zambia - Ndola 2008	35,887	85	91.9%	100.0%	100.0%	100.0%
Zanzibar Kivunge 2002	20,625	148	95.7%	100.0%	100.0%	100.0%
Zanzibar Micheweni 2002	18,236	54	95.2%	99.7%	100.0%	100.0%
Total	23,596	2355	93.9%	99.9%	100.0%	100.0%

### Predicted time of parasite clearance

The number of patients included in RCT at 17 sites with complete parasitaemia record (every 24 hours from Day 0 to Day 3 plus Day 7) was 4,848 of whom 2,355 were treated with ASAQ and 2,493 with a comparator drug.

Using logistic regression, the predicted time of parasite clearance was overall ≈ 31 hours for a median baseline parasitaemia of 27,125/μL, ranging from ≈ 19 hours for patients with parasitaemia before treatment <2,500/μL to ≈ 37 hours for patients with parasitaemia >100,000/μL (Table 5).

**Table 5:** Predicted parasite clearance time by groups of parasitaemia before treatment and intervals, artesunate/amodiaquine treatment

**Parasitaemia at enrolment (μL)**	**N**	**Median**		**Observed**				**pPCT**		
			**Day 0**	**Day 1**	**Day 2**	**Day 3**	**Day 7**	**hour**	**Lower bound**	**Upper bound**
<2,500	110	2,030	100%	24%	2%	1%	0%	19.0	NA	NA
2,500-5,000	227	3,680	100%	44%	7%	1%	0%	24.4	21.7	26.7
5,000-10,000	294	7,290	100%	58%	10%	1%	0%	28.2	23.8	32.5
10,000-20,000	361	14,261	100%	65%	6%	1%	0%	29.1	25.1	36.2
20,000-30,000	261	25,044	100%	68%	10%	0%	0%	30.8	25.7	35.8
30,000-50,000	362	39,250	100%	73%	9%	1%	0%	31.6	27.2	37.1
50,000-75,000	252	61,372	100%	75%	12%	2%	0%	32.9	26.8	37.5
75,000-100,000	159	86,925	100%	79%	13%	0%	0%	34.0	29.8	39.9
>100,000	329	144,357	100%	84%	18%	3%	1%	37.3	33.1	40.7
TOTAL	2,355	27,125	100%	67%	10%	1%	0%	30.8	25.8	41.8

Legend: pPCT, predicted parasite clearance time.

**Figure 3:**
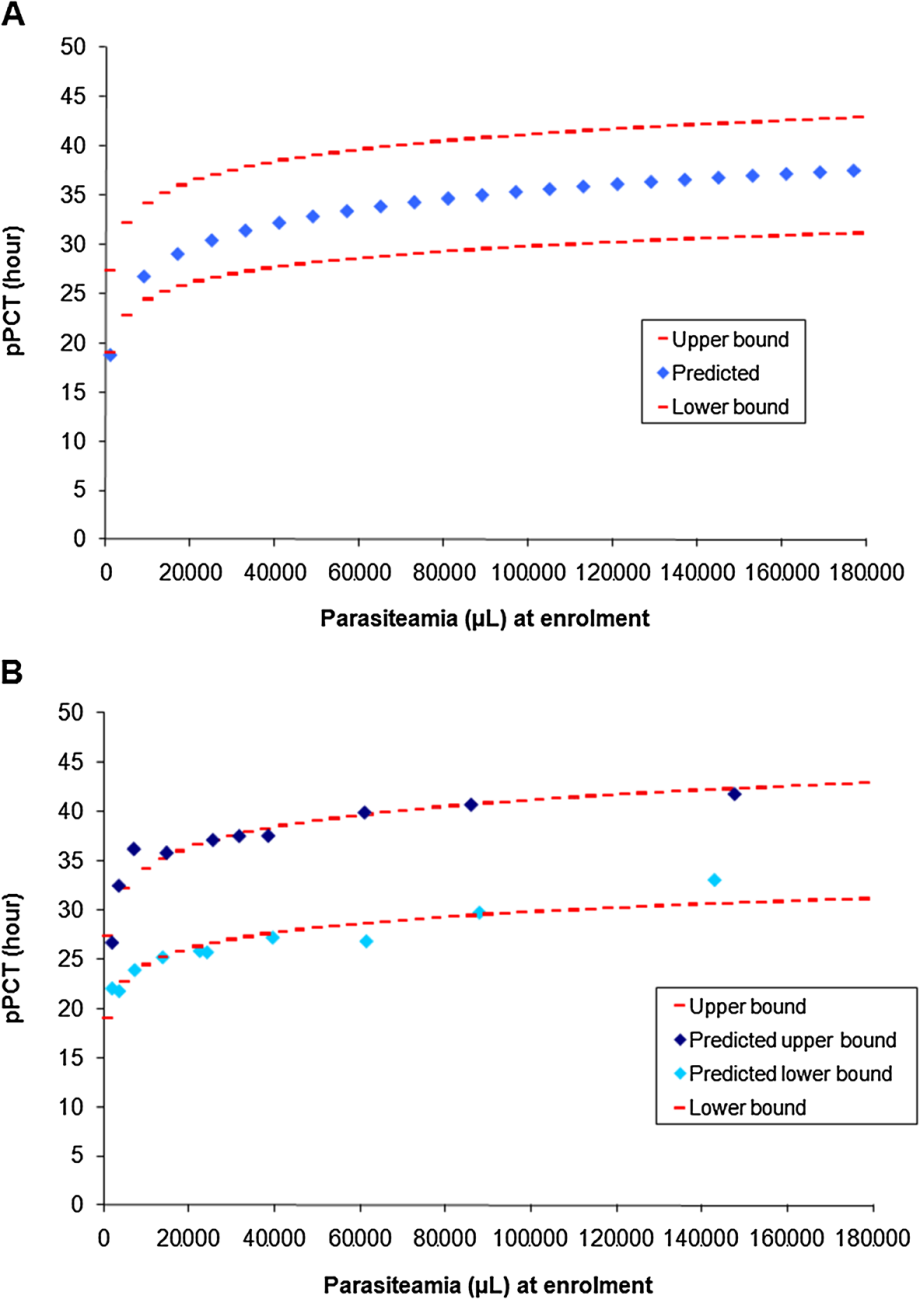
**Fitted curve of parasite clearance and intervals, artesunate/amodiaquine treatment. A**: pPCT, predicted parasite clearance time. **B**: pPCT, predicted parasite clearance time.

Using the predicted PCT from the ten categories of levels of baseline parasitaemia, a logarithmic relationship was detected between the observed median baseline parasitaemia and the corresponding predicted PCT (Figure 3A). The fitted curve was a logarithmic relationship defined by:

$$\begin{array}{l} \;\text{pPCT}=3.614*\ln\left(p0\right)–6.135;r^{2}=0.94\\ \;\text{where}:\text{pPCT}=\text{predicted}\;\text{parasite}\;\text{clearance}\\ \text{time}\;\text{expressed}\;\text{in}\;\text{hours};\text{and}\;p0=\text{parasitaemia}\\ \text{at}\;\text{enrolment}\;(\text{pre}-\text{treatment}) \end{array}$$

The lower and upper bounds of the interval were fitted using sites where the overall proportion of parasitaemic patients were <10% on Day 2 for the lower bound and >10% for the upper bound with a coefficient of determination (r^2^) of 0.88 for both adjustments (Figure 3B). The 10% threshold was in keeping with WHO recommendations [41].

## Discussion

This individual patient data analysis of parasite clearance was conducted on a large sample (n ~15,000) of uncomplicated malaria treatments (predominantly paediatric; three-quarters less than six years old) from 44 sites and 20 sub-Saharan African countries with different levels of endemicity, covering the decade 1999–2009.

This analysis shows that rapid *Plasmodium falciparum* clearance continues to be achieved in sub-Saharan African patients treated with ACT, and in particular with ASAQ, although direct within-site comparisons are not available; it has therefore benchmarking value as reference for future studies. This study also presents a prediction formula for PCT as a pragmatic tool adapted for studies with binary data (not quantitating parasite densities) and once-daily sampling, such as those that would be done at the typical study site in peripheral settings and in routine surveillance by control programmes. This prediction could apply to groups of patients as well as to the individual patient.

These results confirm the correlation between pre-treatment parasitaemia and PCT [10], and offer an easy way to calculate the typical PCT (in hour) using parasitaemia before treatment (p0) with ASAQ or other equivalent treatments (pPCT = 3.614*ln(p0) – 6.135, r^2^ = 0.94). There was also a (weaker) correlation between parasitaemia before treatment and the parasite reduction ratio (PRR) at 24 h.

Monitoring treatment response and detecting artemisinin resistance as early as possible have become a major issue in malaria control. The rate at which treatment clears parasites within the first few days is at present the most useful practical test for ACT, as early response to treatment relies predominantly on the parasite response to artemisinin, independent of whether parasites are later cleared for good through the combination of the longer-lived companion drug and the host’s immune response.

The present analysis focused on initial response to ACT treatment, as a proxy for artemisinin resistance and failure, and aimed to identify variables that were independently associated with persistent parasitaemia on Days 2 and 3 in settings where artemisinin resistance has probably not yet emerged.

Within the period covered by these studies, there was no indication of delayed response to ACT. ACT cleared parasites rapidly leaving very few patients with a low parasitaemia on Day 3 (first day after the completion of the three-day ACT regimen). In particular, with ASAQ the parasite reduction ratio was 93.9% by Day 1 and 99.9% by Day 2; only 1.5% of the patients were still positive on Day 3 (against a proposed threshold of 3% indicating delayed response [10]). These data confirm that pre-treatment parasitaemia is a risk factor for failing to clear parasites on Day 2 and 3. They also show a clear contribution of artemisinins to the speed of action: ACT performed significantly better than non-ACT (whether combinations or single-agent treatments); no difference was detected between ACT (whether the artemisinin derivative was artesunate, artemether or dihydroartemisinin).

Anaemia was an additional risk factor for failing to clear parasites by Day 2; younger patient’s age for Day 3. The only exception was the study conducted in DRC-Boende (Cohuet, unpublished) which had a high proportion of parasite clearance failures with both ASAQ and AL. These findings are difficult to explain and should be taken with caution, but one cannot exclude that there might also have been a focus of resistant *Plasmodium falciparum* in the DRC. No subsequent studies are available to confirm or belie this original study. Understanding the dynamics of parasite clearance could help detect early signs of artemisinin resistance and distinguish biologically meaningful changes in early parasite clearance over time from changes that may be due to the role of effect modifiers like higher pre-treatment parasitaemia, young age and anaemia by applying multivariate analysis.

Consistently with previous results [10] and the first-order process of parasite clearance, there was a logarithmic correlation between patients’ pre-treatment parasitaemia and their pPCT; while developed using ASAQ data, it was applied satisfactorily to other ACT within this database as well as other studies. For example, in a study conducted in Mali [42] where parasitaemia was tested with frequent screenings (every eight hours) in patients treated for uncomplicated P. *falciparum* with artesunate for seven days and where there was no evidence of delayed parasitaemia, the median parasitaemia at enrolment (P0) was 27,070 μL and the observed median time of parasite clearance was 32 hours. Applying the suggested method using the logarithmic correlation found in the present paper (pPCT = 3.614*ln(P0) – 6.135) gave very similar results (30.8 hours with intervals of 26.8 to 37.3 hours).

This simple method was tested to predict a “typical” PCT in situations when resistance has not emerged. However, this estimation of the predicted time to parasite clearance is not without its limitations. The calculations may lack precision with studies sampling once a day and at variable intervals from treatment intake (as it might be the case for most clinical trials, especially on the first day). It has been suggested that parasite clearance rate can only be accurately estimated if sampling is at least every six hours [43]; it is thus possible that data could have been “over-analysed” in generating predicted time of parasite clearance in this case. Although parasite clearance half-life is the research reference to define slow parasite clearance, sampling patients four times or even twice a day is not feasible in routine monitoring, and might be cumbersome even in research settings. Detecting the first signs of artemisinin resistance in uncomplicated *P. falciparum* will likely require a combination of more sophisticated studies and routine monitoring. Between parasite clearance estimators [9] and Day 3 persistency [10], simple estimations of PCT based on daily sampling may add elements to facilitate comparisons and detection of trends. It would be useful to compare methods for validation, including databases with delayed responses.

## Competing interests

The authors declare that they have no competing interests.

## Authors’ contributions

JZ and PO designed the analysis, interpreted the data and prepared the manuscript. All authors read and approved the final manuscript.

## Supplementary Material

Additional file 1: Table S1 Proportion by site of patients still parasitaemic on Day 1, Day 2, Day 3, and parasite clearance failure, ASAQ groups. Click here for file
