# Supplementary material for: Plasmodium falciparum clearance in clinical studies of artesunate-amodiaquine and comparator treatments in sub-Saharan Africa, 1999–2009
Source: Malar J. 2014 Mar 25;13:114. doi: 10.1186/1475-2875-13-114 (PMC3987158; doi:10.1186/1475-2875-13-114)
Supplement: Additional file 1: Table S1 — Proportion by site of patients still parasitaemic on Day 1, Day 2, Day 3, and parasite clearance failure, ASAQ groups. [file 1475-2875-13-114-S1.docx]

# Additional file

Additional table 1: Proportion by site of patients still parasitaemic on Day 1, Day 2, Day 3, and parasite clearance failure, ASAQ groups

| Country, site, year | Day 0 | Day 1 | | | Day 2 | | | Day 3 | | | Total parasite clearance failure (Day 7) | | |
| --- | --- | --- | --- | --- | --- | --- | --- | --- | --- | --- | --- | --- | --- |
|  | Total | Total | Positive slide | | Total | Positive slide | | Total | Positive slide | | Total |  |  |
|  | n | n | n | % | n | n | % | n | n | % |  | n | % |
| Angola-Caala 2004 | 69 |  |  |  | 65 | 1 | 1.5% | 65 | 1 | 1.5% | 65 | 0 | 0.0% |
| Angola-Kuito 2003 | 97 |  |  |  | 97 | 13 | 13.4% | 97 | 5 | 5.2% | 97 | 0 | 0.0% |
| Burkina faso - Nanoro 2008 | 295 | 295 | 171 | 58% | 295 | 3 | 1.0% | 295 | 0 | 0% | 295 | 0 | 0.0% |
| Burkina Faso Puytenga 2005 | 890 |  |  |  | 821 | 21 | 2.6% | 821 | 3 | 0% | 821 | 0 | 0.0% |
| Cameroon 2006 | 110 | 108 | 79 | 73% | 110 | 9 | 8.2% | 110 | 0 | 0% | 110 | 0 | 0.0% |
| Congo-Kindamba 2004 | 101 |  |  |  | 101 | 1 | 1.0% | 101 | 0 | 0% | 101 | 0 | 0.0% |
| Gabon - Fougamou 2008 | 80 | 76 | 37 | 49% | 77 | 1 | 1.3% | 77 | 0 | 0% | 77 | 0 | 0.0% |
| Gabon 1999 | 110 | 106 | 51 | 48% | 104 | 8 | 7.7% | 104 | 0 | 0% | 104 | 0 | 0.0% |
| Guinee-Dabola 2004 | 110 |  |  |  | 110 | 23 | 20.9% | 110 | 5 | 4.5% | 110 | 0 | 0.0% |
| Kenya 1999 | 200 | 198 | 152 | 77% | 197 | 27 | 13.7% | 197 | 3 | 1.5% | 197 | 0 | 0.0% |
| Kenya 2009 | 54 | 53 | 18 | 34% | 53 | 2 | 3.8% | 53 | 0 | 0% | 53 | 0 | 0.0% |
| Liberia 2009 | 645 |  |  |  | 642 | 19 | 2.9% | 148 | 0 | 0% | 146 | 0 | 0.0% |
| Madagascar 2006 | 119 | 118 | 44 | 37% | 118 | 0 | 2.5% | 118 | 0 | 0% | 118 | 0 | 0.0% |
| Mali Bancouna 2004 | 252 | 251 | 174 | 69% |  |  |  | 251 | 1 | 0.4% | 251 | 0 | 0.0% |
| Mali Bougoula 2006 | 135 | 133 | 124 | 93% | 135 | 20 | 14.8% | 135 | 1 | 0.7% | 135 | 0 | 0.0% |
| Mozambique - Manhica 2008 | 210 | 203 | 187 | 92% | 210 | 32 | 15.2% | 210 | 2 | 1.0% | 210 | 0 | 0.0% |
| Nigeria - Afokang 2008 | 92 | 92 | 84 | 91% | 92 | 24 | 26.1% | 92 | 3 | 3.3% | 92 | 0 | 0.0% |
| Nigeria - Pamol 2008 | 82 | 82 | 77 | 94% | 82 | 30 | 36.6% | 82 | 6 | 7.3% | 82 | 0 | 0.0% |
| RDC Boende 2003 | 136 | 47 | 38 | 81% | 133 | 76 | 57.1% | 133 | 44 | 33.1% | 133 | 12 | 8.8% |
| Rwanda Mashesha 2002 | 61 |  |  |  |  |  |  | 61 | 0 | 0.0% | 61 | 0 | 0.0% |
| Rwanda Mashesha 2004 | 89 |  |  |  | 89 | 12 | 13.5% | 89 | 1 | 1.1% | 89 | 0 | 0.0% |
| Rwanda Rukara 2002 | 49 |  |  |  |  |  |  | 49 | 0 | 0% | 49 | 0 | 0.0% |
| Rwanda Rukara 2004 | 89 |  |  |  | 89 | 12 | 13.5% | 89 | 0 | 0% | 89 | 0 | 0.0% |
| Rwanda-Kicukiro 2002 | 48 |  |  |  |  |  |  | 48 | 1 | 2.1% | 48 | 0 | 0.0% |
| Rwanda-Kicukiro 2004 | 74 |  |  |  | 74 | 5 | 6.8% | 74 | 1 | 1.4% | 74 | 1 | 1.4% |
| Sen-Djembeye 2000-5 | 137 | 136 | 94 | 69% | 137 | 12 | 8.8% | 137 | 1 | 0.7% | 137 | 0 | 0.0% |
| Sen-Mlomp 2000-5 | 723 | 638 | 444 | 70% | 667 | 52 | 7.8% | 722 | 4 | 0.6% | 722 | 0 | 0.0% |
| Sen-Oussouye 2000-5 | 106 | 53 | 15 | 28% | 55 | 3 | 5.5% | 105 | 0 | 0.0% | 102 | 0 | 0.0% |
| Senegal 1999 | 160 | 156 | 126 | 81% | 155 | 41 | 26.5% | 155 | 9 | 5.8% | 155 | 1 | 0.6% |
| Senegal 2006 | 264 | 261 | 145 | 56% | 261 | 18 | 6.9% | 261 | 1 | 0.4% | 261 | 0 | 0.0% |
| Sierra Leone Kailahun 2004 | 126 |  |  |  | 126 | 13 | 10.3% | 126 | 7 | 5.6% | 126 | 0 | 0.0% |
| South Sudan Nuba 2003 | 80 |  |  |  | 80 | 17 | 21.3% | 80 | 1 | 1.3% | 80 | 0 | 0.0% |
| Sudan Malakal 2003 | 134 |  |  |  | 132 | 23 | 17.4% | 132 | 7 | 5.3% | 132 | 0 | 0.0% |
| Uganda - Mbarara 2008 | 160 | 157 | 75 | 48% | 157 | 4 | 2.5% | 157 | 1 | 0.6% | 158 | 0 | 0.0% |
| Uganda Amudat 2003 | 106 |  |  |  | 106 | 2 | 1.9% | 106 | 1 | 0.9% | 106 | 0 | 0.0% |
| Uganda Tororo 2004 | 194 |  |  |  | 194 | 12 | 6.2% | 194 | 0 | 0.0% | 194 | 0 | 0.0% |
| Uganda Tororo 2005 | 204 |  |  |  | 204 | 5 | 2.5% | 204 | 1 | 0.5% | 204 | 0 | 0.0% |
| Uganda-Apac-2004 | 174 |  |  |  | 174 | 3 | 1.7% | 174 | 0 | 0.0% | 174 | 0 | 0.0% |
| Uganda-Arua-2004 | 174 |  |  |  | 174 | 12 | 6.9% | 174 | 2 | 1.1% | 174 | 0 | 0.0% |
| Uganda-Jinja-2003 | 189 |  |  |  | 165 | 16 | 9.7% | 189 | 3 | 1.6% | 189 | 1 | 0.5% |
| Uganda-Kampala-2006 | 242 |  |  |  | 241 | 5 | 2.1% | 242 | 0 | 0% | 242 | 0 | 0.0% |
| Zambia - Ndola 2008 | 85 | 81 | 50 | 62% | 85 | 3 | 3.5% | 85 | 0 | 0% | 85 | 0 | 0.0% |
| Zanzibar Kivunge 2002 | 148 | 147 | 84 | 57% | 148 | 2 | 1.4% | 148 | 0 | 0% | 148 | 0 | 0.0% |
| Zanzibar Micheweni 2002 | 54 | 54 | 52 | 96% | 54 | 16 | 29.6% | 54 | 1 | 1.9% | 54 | 0 | 0.0% |
